# Rapid Identification and Visualization of Jowl Meat Adulteration in Pork Using Hyperspectral Imaging

**DOI:** 10.3390/foods9020154

**Published:** 2020-02-06

**Authors:** Hongzhe Jiang, Fengna Cheng, Minghong Shi

**Affiliations:** College of Mechanical and Electronic Engineering, Nanjing Forestry University, Nanjing 210037, China; cfn1218@163.com (F.C.); mhshi@njfu.edu.cn (M.S.)

**Keywords:** hyperspectral imaging, jowl meat, minced pork, meat adulteration, visualization

## Abstract

Minced pork jowl meat, also called the sticking-piece, is commonly used to be adulterated in minced pork, which influences the overall product quality and safety. In this study, hyperspectral imaging (HSI) methodology was proposed to identify and visualize this kind of meat adulteration. A total of 176 hyperspectral images were acquired from adulterated meat samples in the range of 0%–100% (w/w) at 10% increments using a visible and near-infrared (400–1000 nm) HSI system in reflectance mode. Mean spectra were extracted from the regions of interests (ROIs) and represented each sample accordingly. The performance comparison of established partial least square regression (PLSR) models showed that spectra pretreated by standard normal variate (SNV) performed best with R_p_^2^ = 0.9549 and residual predictive deviation (RPD) = 4.54. Furthermore, functional wavelengths related to adulteration identification were individually selected using methods of principal component (PC) loadings, two-dimensional correlation spectroscopy (2D-COS), and regression coefficients (RC). After that, the multispectral RC-PLSR model exhibited the most satisfactory results in prediction set that R_p_^2^ was 0.9063, RPD was 2.30, and the limit of detection (LOD) was 6.50%. Spatial distribution was visualized based on the preferred model, and adulteration levels were clearly discernible. Lastly, the visualization was further verified that prediction results well matched the known distribution in samples. Overall, HSI was tested to be a promising methodology for detecting and visualizing minced jowl meat in pork.

## 1. Introduction

Meat always plays an important role in the constitution of human diets around the world. People consume meat mainly due to its rich nutritional contents of essential amino acid and vitamins [1]. With the continuous and rapid increase of meat consumption nowadays, meat quality and safety control has become the major priority [2]. Meat is quite susceptible to suffering adulteration, such as the known horsemeat scandal in 2013 that horsemeat was detected in beef. From the perspective of consumers, they highly desire reliable and clear information about the meat or meat products they purchase [3]. Therefore, regardless of deliberate, accidental, or economically motivated meat adulteration, rapid identification is always one of the main issues in further prevention.

Minced meat, also named ground meat, is one of the most popular meat types. It is versatile in that it can be the major ingredient of a variety of meat products including sausages, patties, hamburgers, and meatballs [4]. Especially in Chinese diet culture, traditional dumplings, steamed stuffed buns, or wontons fillings are also mainly composed of this important ingredient. However, the morphological structure of muscles is removed when meat is minced so that the occurrence of adulteration in minced meat is hardly recognized by visual analysis. Pork jowl meat is a kind of lymphatic meat, which contains thyroid, lipomas, and significant amounts of lymph nodes [5]. It is low-price, full of stench, and should be discarded after the animals are slaughtered [5]. In the present period, a relatively high rate of fraudulent phenomena occur in China involving minced jowl meat being substituted for or added into minced pork to be served as fillings in food. These incidents seriously pose health treats and violate rights for consumers. Meat adulteration is not routinely detected, thus, it is highly desirable to rapidly identify if the fillings are adulterated with jowl meat.

Several detection techniques including high-performance liquid chromatography [6], polymerase chain reaction [7], mass spectrometry [8], differential scanning calorimetry [9], and enzyme-linked immunosorbent assays [10] were demonstrated to be effective in detecting meat adulteration. However, these techniques need complex sample preparation and destructive operation, which are high-cost, time-consuming, and laborious. More recently, a considerable number of fast optical techniques have shown potential in detecting meat adulteration. Among them, UV-visible [11], near-infrared spectroscopy (NIRS) [12,13], mid-infrared spectroscopy [14], Raman spectroscopy [15], laser induced breakdown spectroscopy [16], and computer vision [17] have been successfully developed to identify various adulterants in meat. Typically, conventional NIRS could overcome the above-mentioned drawbacks of traditional techniques for its sensitive, rapid, reagent-free, and nondestructive nature, and it has great potential to adapt real-time monitoring application [18,19]. However, the main limitation is that the spectral single-point detection in preselected areas cannot well represent the whole heterogeneous meat sample. In this light, interest in hyperspectral imaging (HSI) is continuously growing. HSI integrates conventional spectroscopy and imaging to acquire both spectral and spatial information from an identical sample to provide the traceable chemical and physical qualities simultaneously [20]. It has received a lot of attention and interest in inspecting both raw and processed meat items [21,22].

With regard to the detection of meat adulteration using HSI, several examples have previously been reported, for instance, pork or beef adulterated with chicken [17], chicken adulterated with carrageenan [23], beef adulterated with pork [24,25], prawn adulterated with gelatin [26], lamb adulterated with pork [27], beef adulterated with horsemeat [28,29], lamb adulterated with duck [30], etc. All the above studies achieved good performance through a combination of HSI and chemometrics. However, to date, few studies focused on the adulteration of minced pork with minced jowl meat.

Therefore, the main aim of this study is to investigate the feasibility of visible and near-infrared (VNIR, 400–1000 nm) HSI for detecting minced jowl meat in pork. The specific objectives were (1) to establish partial least square regression (PLSR) models based on spectra extracted from hyperspectral images and (2) to identify effective wavelengths for developing multispectral models and visualizing the adulteration.

## 2. Materials and Methods

### 2.1. Sample Preparation

Pure pork meat (from *Longissimus dorsi* muscle) and adulterant of jowl meat from the homologous slaughtered porcine body were purchased from a local retail market in Nanjing, China and transported to our laboratory within 30 min. On the day of purchase, pork meat was first cut into small pieces and minced using a meat grinder (S2-A808, Joyoung Co. Ltd., Jinan, China) for 60 s. The grinder was carefully washed using detergent and hot water, rinsed with distilled water, wiped with paper towel, and totally dried before jowl meat mincing use. In the same way, jowl meat was subsequently minced to be used as the adulterant. Minced pork samples were adulterated by mixing the minced adulterant into pork in range of 10%–90% (w/w) at 10% increments. Additionally, pure pork (0% adulteration level) and pure jowl meat (100% adulteration level) samples were also prepared. Sixteen replicates were prepared for each adulteration level, and samples were individually weighted. They were thoroughly mixed together to obtain a roughly homogenous paste with a final weight constant at 50 g. Then, all the prepared samples were put into round disposable Petri dishes (9 cm in diameter × 1.4 cm deep) with flat surfaces for subsequent imaging procedure.

In total, 176 samples including 16 samples at each adulteration level (11 levels) were prepared. Among them, three quarters of the samples at each adulteration level (12 samples × 11 levels = 132 samples) were randomly selected to be assigned to calibration set, and the residual one quarter (4 samples × 11 levels = 44 samples) were used for purpose of independent prediction. Furthermore, in order to prove the creditability of visualization results, two more control samples with known distributed patterns were also prepared. As for control sample one, four fan-shaped parts at different individual adulteration levels of 100%, 80%, 40%, and 20% were included. With regard to control sample two, two semicircular areas at individual 0% and 50% adulteration levels were covered. After that, the hyperspectral images of all the prepared samples were subsequently captured.

### 2.2. Hyperspectral Image Acquisition and Calibration

All the prepared samples were scanned using a laboratory-based push-broom hyperspectral imaging system in reflectance mode. The system converted the visible and near-infrared (VNIR) spectral range of 400–1000 nm (284 spectral bands) to capture the hyperspectral images at room temperature (26 ± 1 °C). The system consisted of a computer (Lenovo Tianyi 510 Pro, Lenovo Group Ltd., Beijing, China) installed with data acquisition software (Spectral Image software, Isuzu Optics Corp., Taiwan, China), a spectrograph (ImSpectorV10E, Spectral Imaging Ltd., Oulu, Finland), a 12-bit charged couple device (CCD) camera (HScamera-VIS, Isuzu Optics Corp., Xinzhu, China) with a C-mount lens, an illumination unit of two 150-W tungsten-halogen lamps, and a translation stage (Specim Spectral Imaging Ltd., Oulu, Finland) powered by stepping motor (SC30021A, Zolix Instrument Co, Beijing, China). The spectral solution of the system was 2.8 nm, and the size of incident slit was 30 μm (width) × 14.2 mm (length).

After hyperspectral image acquisition, calibration was conducted using two reference images by the equation as follows:

(1) $$R_{c}\;=(R_{o}-\;D)/(W\;-\;D)\;\times \;100\%$$

where R_c_ represents calibrated relative reflectance hyperspectral image, R_o_ denotes acquired original hyperspectral image, D expresses dark reference hyperspectral image with about 0% reflectance, and W stands for white reference hyperspectral image with about 99.9% reflectance. This procedure was directly carried out through the images calibration function within data acquisition software.

### 2.3. Region of Interests (ROI) Identification and Spectral Extraction

To isolate pure meat portion in the acquired images, representative regions of interests (ROIs) were first taken based on the calibrated images. ROI was individually determined for each hyperspectral image by applying a corresponding binary mask which was first established using band math procedure. Initially, the low reflectance image at 450 nm was subtracted from the high reflectance image at 890 nm. ROI was formed within the resulting image by thresholding at a constant value of 0.2. The ROIs were completely isolated from backgrounds and edges of the Petri dishes. After that, the mask was built and applied to corresponding hyperspectral images. Then, average spectra were extracted to represent each corresponding sample. All the involved steps were conducted using functions of mask building and ROI generation in ENVI 5.3 (Research Systems Inc., Solutions, Boulder, CO, USA).

### 2.4. Spectral Pretreatments

In order to compare and obtain robust and reliable performance, spectral data pretreatment is necessary prior to the development of quantitative models. In this research, a series of pretreatments including normalization, standard normal variate (SNV), multiplicative signal correction (MSC), detrending (detrend), first-order derivative, and second-order derivative (1st and 2nd derivative) were applied in addition to non-preprocessed spectra. Multiplicative interferences of scatter in the spectra can be effectively removed using SNV approach. MSC is a method like SNV which is effectively used in multiplicative variations elimination. Derivatives were usually used to remove baseline offsets and separate overlapping absorption bands. In this research, derivatives were calculated using second-order polynomial with Savitzky–Golay smoothing by a moving window size of 15 points. Detrending was implemented combined with SNV to suppress the baseline shifting and curvilinearity. Normalization was utilized to present the spectral differences caused by slight optical path variations. The pretreatment or the combinations were all implemented in the Unscrambler X 10.1 (Camo Software Inc., Trondheim, Norway).

### 2.5. Modeling Method

PLSR is a reliable linear regression modeling method that has been widely employed in spectral analysis for quantitatively predicting agro-products’ quality traits. This method is especially suitable in performing the situation where there is a linear relationship between attributes of the targets and variables. In this study, PLSR models were developed with the dataset in calibration set using a leave-one-out cross-validation (LOOCV) method to prevent data over-fitting. PLSR first projects the spectra onto a few orthogonal factors named latent variables (LVs) [31]. The optimal LVs were determined where lowest root mean square error value of cross-validation was achieved. The PLSR modeling procedure was carried out using the software MATLAB 2013b (MathWorks Inc., Natick, MA, USA) with the PLS toolbox.

### 2.6. Wavelengths Selection Methods

Principal component analysis (PCA) is an unsupervised exploratory technique that has been reported to be a powerful tool in dimensionality reduction and multivariate data visualization. The variances of the whole dataset are first explained by PCA, and only a few new orthogonal latent variables that maximize the data variance (called the principal components: PCs) are retained [32]. PCA helps to look for relationships among the samples, and samples in the same class will gather together in PC score plots. If a clear clustering of grouped samples is shown in the PC space by combining two or more PCs, corresponding PC loadings are effective to determine informative wavelengths. Then, pronounced peaks and valleys of the PC loadings are considered to contribute more to the spectral variations of samples with different adulteration levels. The PCA procedure was conducted using the MATLAB 2013b.

Two-dimensional correlation spectroscopy (2D-COS) is a commonly used analytical mathematical formalism. Recently, 2D-COS analysis is highly concerned with identifying a set of spectroscopic data including Raman, visible-infrared, and fluorescence under external perturbation [33]. In terms of generalized 2D-COS, the perturbation can be pressure, concentration, temperature, etc. [34]. To discuss the generated spectrum, synchronous spectrum is diagonal symmetry and there were several autopeaks located at diagonal line. The synchronous spectrum could be used to characterize differences of the spectral intensities at different wavelengths. If the spectral intensities changed sharply with levels at a certain wavelength, there will be a strong autopeak. Thus, in our study, the autopeaks were introduced and utilized as effective wavelengths for identifying the adulteration.

Regression coefficients (RC), which are always used in combination with PLSR modeling method, are also an effective wavelength selection approach. In PLSR models, peaks and valleys at certain wavelengths with dominated RC values indicate a high influence on the response (predicted results) [35]. These spectral variables would be more useful in PLSR modeling and should be chosen for further PLSR models simplification. In our study, wavelengths with high absolute RC values (above the cutoff threshold) from the optimal PLSR model were considered to contribute most in predicting adulteration levels and they were finally adopted.

### 2.7. Models Performance Assessment

In order to assess the performance of established models, the following criteria including coefficient of determination in calibration set (R_c_^2^), cross-validation (R_cv_^2^), and prediction sets (R_p_^2^), as well as root mean squared error in calibration (RMSEC), cross-validation (RMSECV), and prediction sets (RMSEP) were determined, respectively. Furthermore, residual predictive deviation (RPD) was also evaluated to assess the practical utility of prediction models. If the values of R^2^ ≥ 0.70 and RPD ≥ 2.00, models were considered to be effective in detecting meat quality and safety [36]. A satisfactory model should perform with results of high values in R_c_^2^, R_cv_^2^, R_p_^2^, and RPD as well as low values in RMSEC, RMSECV, and RMSEP.

### 2.8. Distribution Maps of the Adulterant

Recognizing adulterate distribution in minced pork is helpful to rapidly observe general adulteration level or if the sample was adulterated. The generation of distribution map is a means of visualization. It is a special advantage of HSI that conventional imaging or NIRS could not achieve. The optimal simplified model can be applied back to predict values in each pixel in the multispectral images at selected wavelengths. After that, the distribution map which pieced all predicted values of pixels together is generated. Therefore, in this research, after the optimal simplified model was confirmed, a colorful image with a linear color scale used for visualizing adulteration levels was displayed. All these steps were performed using a homemade program developed in Matlab 2013b.

## 3. Results and Discussion

### 3.1. Spectral Profiles

**Figure 1:**
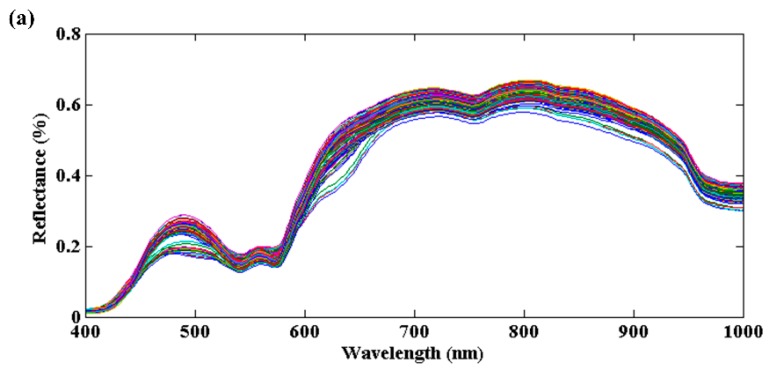

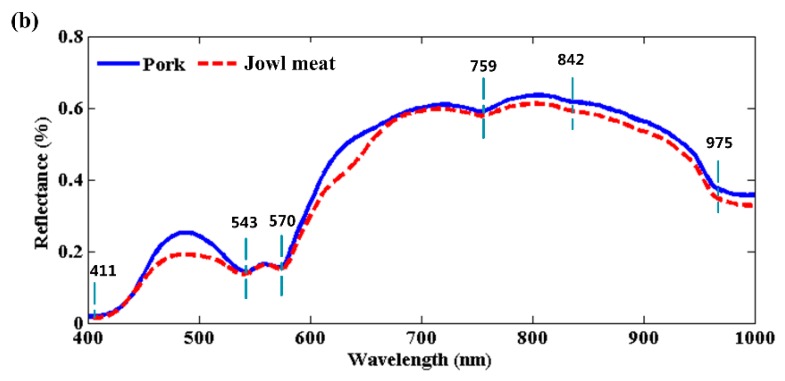
Spectral characteristics of prepared samples in the visible and near-infrared region. (**a**) Adulterated samples, (**b**) Mean raw spectra of pure pork and jowl meat.

The average reflectance spectra of all the adulterated samples (Figure 1a) and pure minced pork and jowl meat extracted from the corresponding hyperspectral images (Figure 1b) are presented in Figure 1. Different spectra showed similar patterns with certain differences in reflective intensity. As shown, minced pork samples had slightly higher reflective intensity than minced jowl meat samples in spectral region of 400–1000 nm. Although there were few overlaps between spectra of pork and jowl meat, it was still possible to observe certain spectral differences, especially at prominent peaks or valleys. The variations in spectral reflectance among pork and jowl meat were related to the differences in chemical composition, and this implies the adulteration would induce significant alterations to the pure pork samples in a way that can be detected using spectral information.

In the VNIR region, several downwards peaks (absorbance bands) could be observed for pork and jowl meat. The band centered at around 411 nm was associated with the Soret absorption band, which was due to a respiratory pigment of haemoglobin [37]. The valleys of 543 nm and 570 nm can be ascribed to the presentence of deoxymyoglobin and oxymyoglobin, respectively [38], which were responsible for color traits of meat. The 975 nm and 759 nm were attributed to the second and third overtone of O–H stretching mode of water, respectively [39,40]. As for a weak reflectance valley, the independent absorbance band of 842 nm could correspond to the C-H stretching mode of aliphatic compounds [41,42]. The comparison showed that the reflective differences at these bands indicated that minced jowl meat samples had more contents of myoglobin, fat, and water than pure pork. Thus, the identification of jowl meat adulteration in pork is preliminarily concluded to be feasible on the basis of its spectral characteristics difference.

### 3.2. PLSR Models

To determine and quantify the adulteration levels, PLSR models were developed based on raw or pretreated (normalization, SNV, MSC, SNV + Detrend, 1st derivative and 2nd derivative) spectra. A summary of the predictive results is listed in Table 1. As can be seen, spectral data with or without various pretreatments all showed good capability in predicting the adulteration levels. The overall R^2^ values were higher than 0.94, RMSE values were lower than 10.2%, and RPD values were higher than 3.1. The small differences observed among RMSEC, RMSECV, and RMSEP values indicated that models were robust and reliable. Overall, HSI coupled with PLSR modeling method provided an innovative way to perform the instant and noncontact prediction for jowl meat adulterated in pork. With regard to a comparison of different pretreatments, the best PLSR modeling results were obtained based on SNV pretreated spectral data with performance of R_p_^2^ = 0.9549, RMSEP = 7.04%, and RPD = 4.54. Thus, the pretreatment of SNV was adopted to preprocess spectra in subsequent analysis of model simplification and visualization.

### 3.3. Wavelengths Selection

#### 3.3.1. PCA Explanatory Analysis

**Figure 2:**
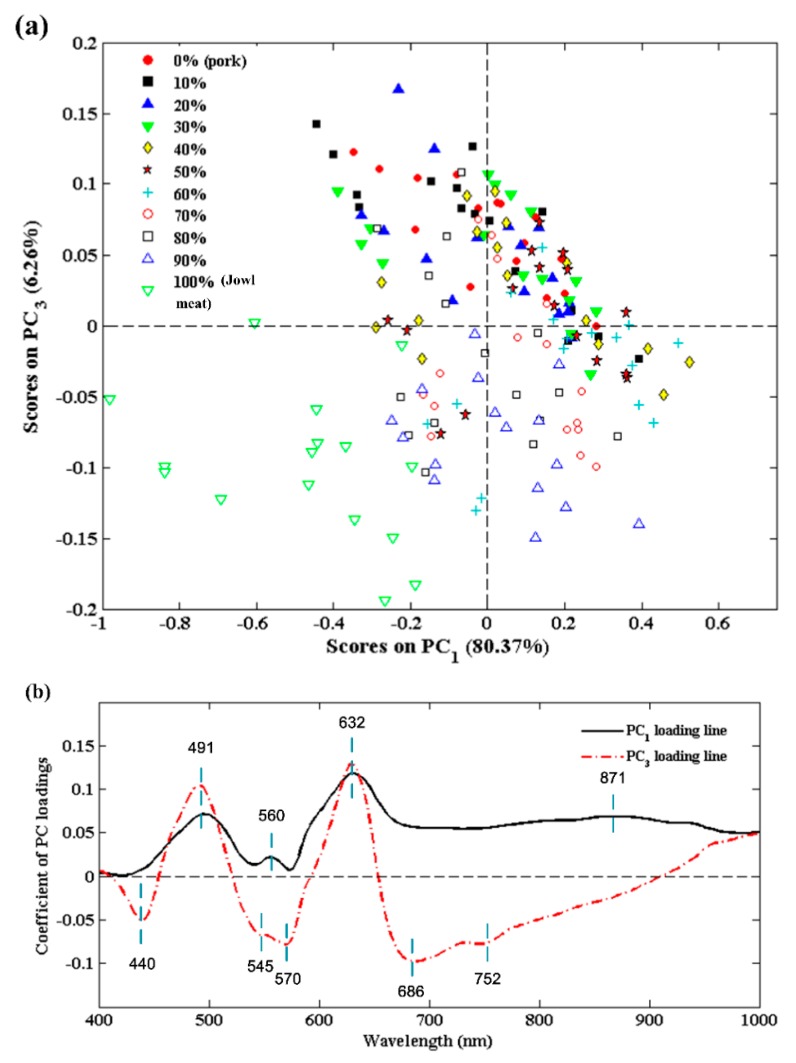
Analysis of effective PC scores and loadings. (**a**) PCA score plot of PC_1_ vs. PC_3_, (**b**) Wavelength selection on PC_1_ and PC_3_ loading lines.

Principal component analysis (PCA) is an efficient chemometric method, which provides the interpretation of variances among different data points in spectral analysis. In order to compare and highlight the spectral similarities and differences, PCA was first applied to the whole dataset of 176 spectra. The first three PCs which individually accounted for 80.37%, 9.50%, and 6.26% of the total variance were retained. The reason was that above 95% of the variation could be explained by the first three PCs. Moreover, through trial and error with different PC combinations, PC_1_ and PC_3_ were found to be useful in grouping samples into different adulteration levels. Then, the calculated PC scores of data points for different adulteration levels were utilized to create a 2D score plot (Figure 2). In general, data points in the same adulteration level tended to gather together and will be separated from others. The score plot of the combination of PC_1_ vs. PC_3_ is shown in Figure 2a. As the adulteration level continued to rise, corresponding samples tended to move along the positive directions of PC_1_ axis and PC_3_ axis. However, in low-level adulteration (less than 30%), data clusters were observed to be quite close and overlapped in a certain level. Tracing the root of the above observation, the main chemical composition of homologous pork and jowl meat was too similar so that no distinct separation was displayed if adulteration level was low. In addition, PC_2_ seemed to mainly express the mutual information of samples with different adulteration levels, which was eliminated in the discrimination.

The PC loading lines of the two effective PCs were analyzed in detail and plotted in Figure 2b. Wavelengths at pronounced peaks and valleys were considered to carry important information in identifying the adulteration and should be selected. As a result, a total of nine wavelengths (440 nm, 491 nm, 545 nm, 560 nm, 570 nm, 632 nm, 686 nm, 752 nm, and 871 nm) were chosen. The valley at 440 nm could be attributed to deoxymyoglobin, the peak at 491 nm is associated with metmyoglobin, and 632 nm could be assigned to sulfmyoglobin [38]. The 676 nm is related to the presentation of redness, and the 871 nm band is relevant with the C-H vibration of hydrocarbons. The wavelengths selected by spectral PCA further confirmed the above results in spectral characteristics that pork and jowl meat were different in color presentation as well as water and hydrocarbon contents.

#### 3.3.2. Two-Dimensional Correction Spectroscopy

**Figure 3:**
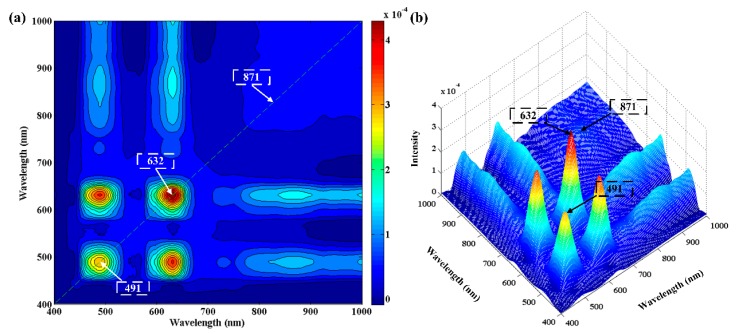
The 2D-COS spectrum of samples with various adulteration levels. (**a**) Synchronous contour map plot, (**b**) Corresponding synchronous 3D stereo plot.

The 2D-COS analysis of the obtained 11 average spectra with adulteration levels from 0% to 100% is shown in Figure 3. There were two dominant autopeaks of 491 nm and 632 nm observed at the diagonal line in synchronous spectrum (Figure 3a). Another weak autopeak of 871 nm also occurred which could be clearly seen in the corresponding 3D stereo plot in Figure 3b. The presence of these suggested that intensity at these bands varied seriously with the adulteration levels. Therefore, these three wavelengths were effective in identifying the adulteration levels. It is worth mentioning that these three selected wavelengths were also included in the wavelengths selected by PC loadings. In terms of spectral variables, these three wavelengths are the most important in identifying the adulteration.

#### 3.3.3. Regression Coefficients

**Figure 4:**
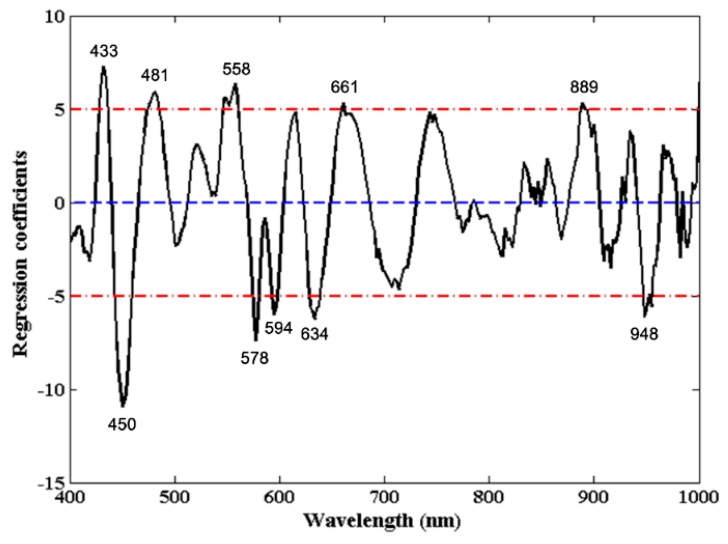
Regression coefficients of the optimal PLSR model based on full spectra.

The regression coefficients (RC) curve from the preferred PLSR model based on SNV pretreated spectra is shown in Figure 4. The cut-off threshold was set to be 5, and only wavelengths at peaks and valleys with higher absolute coefficients than 5 were retained. Finally, a total of 10 (433 nm, 450 nm, 481 nm, 558 nm, 578 nm, 594 nm, 634 nm, 661 nm, 889 nm, and 948 nm) discontinuous wavelengths were deemed as the most effective wavelengths in PLSR models’ development for quantitatively predicting jowl meat adulteration in minced pork. These wavelengths were different from the above ones but also reasonable due to the consideration of targeted prediction values.

Based on the above three wavelengths selection methods, the number of variables reduced significantly by at least 96.5% to at most 98.9%. The retained wavelengths could be utilized in developing a robust model and further a low-cost multispectral imaging system. Therefore, all the three groups of effective wavelengths could be the basis for comparison in developing the simplified PLSR models.

### 3.4. Multispectral Models Development

In order to further eliminate the useless wavelengths and optimize the processing time in computing, simplified PLSR models based on selected wavelengths were established. Wavelengths selected by 2D-COS, PC loadings, and RC methods were individually set as inputs of the simplified PLSR models, and the overall results are displayed in Table 2. As can be seen, the results obtained using selected wavelengths slightly decreased compared with the full spectra. This phenomenon indicated that selected wavelengths were effective and the eliminated variables also contained little information in determining the adulteration. Compared with 2D-COS-PLSR and PC loadings-PLSR models, RC-PLSR model achieved the best performance with R_p_^2^ of 0.9063, RMSEP of 13.93%, and RPD of 2.30. It indicated that the 10 wavelengths selected by RC method were the most critical in identifying jowl meat adulteration in pork. On the contrary, the three wavelengths selected by 2D-COS performed not that well mainly because that they were not informative enough. As an extension, the additional six more wavelengths selected using PC loadings significantly improved the prediction accuracy by showing the R_p_^2^ of 0.7475, RMSEP of 17.31%, and RPD of 1.85. What is more, 2D-COS and PC loadings selected wavelengths using X-variables (spectra) only, while RC was based on PLSR model which decomposed both X-and Y-variables (adulteration levels) in the LVs calculation. RC built the optimal relationship between spectral data and adulteration levels compared with 2D-COS and PC loadings. Therefore, the multispectral RC-PLSR model was finally chosen for further visualization steps.

The determination of the limit of detection (LOD) is an important step which investigated if the lowest adulteration concentration can be detected with the HSI methodology. The LOD was commonly calculated to evaluate the sensitivity of detection methods by the following equation [43].

LOD = 2*δ*_b_/*S* (2)

where *δ*_b_ indicates the standard deviation (SD) of the background response and *S* denotes the sensitivity by the ratio of the predicted adulteration levels to the reference values (namely the slope of the calibration line).

**Figure 5:**
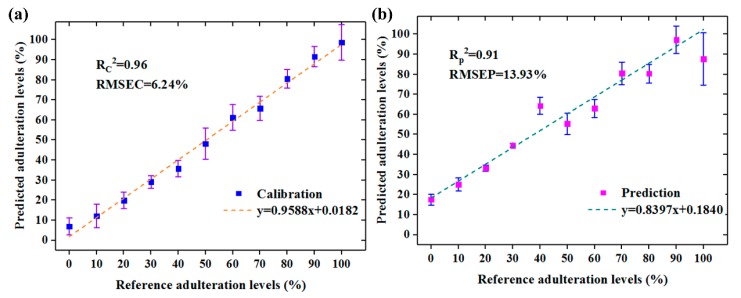
The performance of preferred multispectral RC-PLSR model with error bar in (**a**) Calibration set, and (**b**) Prediction set.

The performance of the preferred multispectral RC-PLSR model with error bar is illustrated in Figure 5. The results of this optimal model were visualized, and it could be seen that all the adulterated samples could be detected (predicted values were above 0%) whether in the calibration or prediction set. The LOD in independent prediction set calculated by the above Equation (2) was found to be 6.50%. However, the aim of meat adulteration is generally to make profit so that adulteration is always performed to be more than 10% [44]. Thus, the LOD in this research proved that it was competent in detecting jowl meat adulteration in pork by the HSI system.

### 3.5. Visualization of the Adulteration Levels

**Figure 6:**
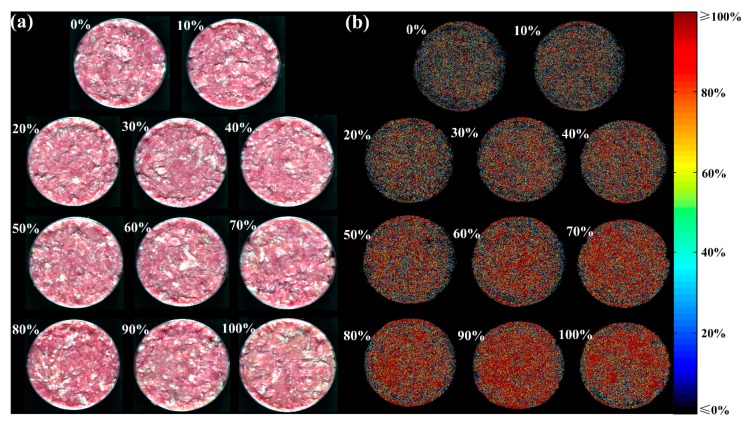
Distribution maps of jowl meat adulteration in pork at pixel level. (**a**) False color images, (**b**) Distribution maps.

As known, each sample was represented by the average spectra extracted from corresponding ROI, and the targeted adulteration level was only indicated by one value. However, there was abundant spatially distributed information in hyperspectral images. In this research, the adulteration level at each pixel in one hyperspectral image was predicted so that distribution was visualized for a quick view. The optimal RC-PLSR model was first applied to the multispectral images recombined at selected wavelengths. The false color images (left column) and corresponding predicted distribution maps (right column) of the samples with different adulteration levels in prediction set are shown in Figure 6. The false color image in Figure 6a was composited by setting the images at 700.9 nm, 545.4 nm, and 436.4 nm as R (red), G (green), and B (blue) channels using ENVI software. They were quite close to the true color image at the primary RGB colors’ wavelengths (700 nm, 546.1 nm and 435.8 nm). As can be seen, actual adulteration level is difficult to be recognized by naked eyes in Figure 6a. Distribution maps expressed how the adulteration varied from sample to sample and even from pixel to pixel within one sample and were generated to be shown in Figure 6b. The linear color bar located in the right side from black to red indicated different adulteration levels from 0% to 100% accordingly. There was a clear tendency of color gradient with the increasing adulteration level so that adulteration was easily distinguishable in distribution maps.

**Figure 7:**
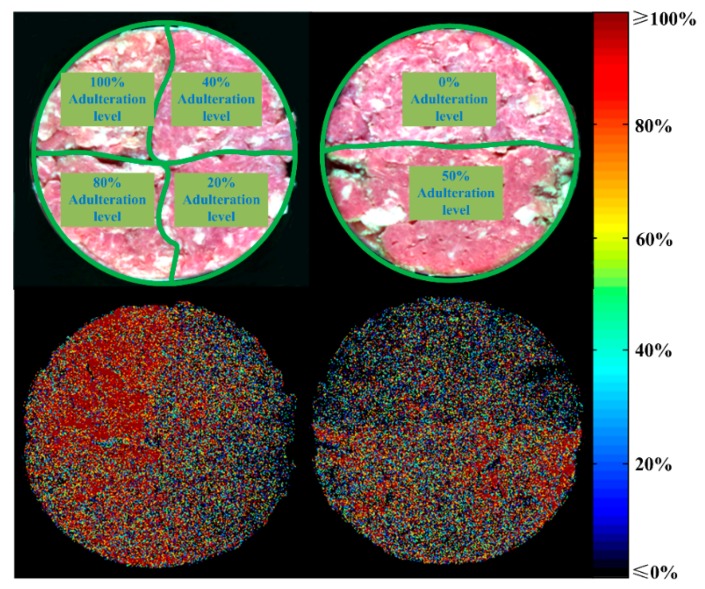
Visualization for control samples.

In further steps, the reliability of visualization was verified through generating distribution maps for the two control samples. Two false color images were in the up row and corresponding distribution maps were displayed in the down row in Figure 7. For control sample one, the results of the distribution map showed that four fan-shaped areas were in different colors. The upper left area was mainly in red, and the lower left showed red and yellow. The upper right exhibited a wide range of colors while the lower right presented general blue. With regard to control sample two, the distribution map gave a display of half black and half yellow. All the prediction maps were generally consistent with the actual situation. The effectiveness of the established RC-PLSR model and visualization procedure was thus proved again.

## 4. Conclusions

This study was motivated by the requirement for rapidly and nondestructively identifying one common adulterant of minced jowl meat in minced pork. Our attempts explored the application of HSI combined with chemometrics and wavelength selection algorithm to quantify and visualize the adulteration. In particular, simplified RC-PLSR model developed by 10 key wavelengths gave the best performance; LOD achieved 6.50%. The visualization of adulteration levels was successfully performed based on RC-PLSR model. Predicted colorful distribution maps were generated to make it convenient in observation. To test the validity of visualization, two known distributed samples were predicted and expected corresponding maps were displayed. The overall results suggested that HSI had the potential to identify minced jowl meat adulteration in pork without any prior physical or chemical analysis information. This technique could provide more detailed visualization information than conventional imaging and NIRS in identifying adulteration levels. However, more studies related to a large number of samples and different breeds in sampling should be conducted. In addition, further work will focus on identifying a variety of commonly used adulterates in pork based on a few wavelengths to achieve the goal for portable application.

## Figures and Tables

**Table 1:** Performance of partial least square regression (PLSR) models for predicting minced jowl meat adulterated in pork.

Pretreatments	LVs	Calibration		Cross-Validation		Prediction		
		R_c_^2^	RMSEC	R_cv_^2^	RMSECV	R_p_^2^	RMSEP	RPD
None	12	0.9866	3.64%	0.9779	4.71%	0.9458	7.50%	4.27
Normalization	14	0.9898	3.18%	0.9821	4.24%	0.9493	7.25%	4.41
SNV	12	0.9864	3.68%	0.9787	4.60%	0.9549	7.04%	4.54
MSC	13	0.9878	3.50%	0.9801	4.45%	0.9536	7.06%	4.53
SNV + Detrend	12	0.9870	3.59%	0.9797	4.51%	0.9512	7.47%	4.28
1st derivative	13	0.9886	3.36%	0.9815	4.30%	0.9528	7.19%	4.45
2nd derivative	14	0.9896	3.23%	0.9797	4.50%	0.9425	10.18%	3.14

Notes: PLSR: partial least squares regression; SNV: standard normal variate; MSC: multiplicative scatter correction; LVs: latent variables; R_c_^2^: coefficient of determination in calibration set; R_cv_^2^: coefficient of determination in cross-validation set; R_p_^2^: coefficient of determination in prediction set; RMSEC: root mean squared error for calibration set; RMSECV: root mean squared error for cross-validation set; RMSEP: root mean squared error for prediction set; RPD: residual predictive deviation.

**Table 2:** Performance of simplified PLSR models based on wavelengths selected by three methods.

Method	Number	LVs	Calibration		Cross-Validation		Prediction		
			R_c_^2^	RMSEC	R_cv_^2^	RMSECV	R_p_^2^	RMSEP	RPD
2D-COS	3	3	0.2283	27.78%	0.1920	28.45%	0.2720	27.45%	1.17
PC loadings	9	6	0.8981	10.09%	0.8344	10.80%	0.7475	17.31%	1.85
RC	10	9	0.9610	6.24%	0.9520	6.93%	0.9063	13.93%	2.30

*Notes:* PLSR: partial least squares regression; 2D-COS: two-dimensional correction spectroscopy; PC: principal component; RC: regression coefficients; LVs: latent variables; R_c_^2^: coefficient of determination in calibration set; R_cv_^2^: coefficient of determination in cross-validation set; R_p_^2^: coefficient of determination in prediction set; RMSEC: root mean squared error for calibration set; RMSECV: root mean squared error for cross-validation set; RMSEP: root mean squared error for prediction set; RPD: residual predictive deviation.
